# The Reliability of the Progression of Autonomies Scale Applied on Acquired Brain Injured Patients

**DOI:** 10.3389/fneur.2019.00342

**Published:** 2019-04-10

**Authors:** Francesco Arcuri, Maria Daniela Cortese, Francesco Riganello, Lucia Francesca Lucca, Sebastiano Serra, Anna Mazzucchi, Antonio Cerasa, Paolo Tonin

**Affiliations:** ^1^Research in Advanced Neurorehabilitation, S. Anna Institute, Crotone, Italy; ^2^Department for ABI Care and Rehabilitation, Don Gnocchi Foundation, Milan, Italy; ^3^Neuroimaging Unit, IBFM-CNR, Catanzaro, Italy

**Keywords:** inter-rater agreement, intra-rater agreement, reliability, autonomies scale, acquired brain injury

## Abstract

The Progression of Autonomies Scale (PAS) is a behavioral scale useful to assess the autonomy levels in acquired brain-injured patients. It provides a broad profile, assessing different domains of human activities ranging from personal, domestic, and extradomestic autonomies. This cross-sectional study is aimed at evaluating the reliability of this scale on a large cohort of acquired brain injury (ABI) patients. Fifty-one ABI patients (49% traumatic, 33.3% hemorrhagic, 17.7% other etiologies), hospitalized in the S. Anna Institute of Crotone, Italy (mean age male 46.08 ± 14.53 and mean age female patients 43.2 ± 11.3) were recruited. We found a high level of reliability of the scale, with a coefficient at the inter-rater agreement between substantial (0.61 ≤ *k* ≤ 0.8) and almost perfect (0.81 ≤ *k* ≤ 1), and almost perfect at the test-retest (intra-rater). We confirm that the PAS is a well-structured tool for the assessment of the autonomy levels in brain-injured patients. These findings encourage the application of this scale in the clinical practice of rehabilitation unit to design a tailored rehabilitation treatment on real goals and to monitor the generalization of the recovered abilities to the daily routine activities.

## Introduction

Recovering participation in self-care, leisure and productivity domains is the ultimate goal in the rehabilitation process of acquired brain injured (ABI) patients. Generally, due to the nature and location of the injury, the clinical picture of ABI patients is characterized by a wide heterogeneity (1). Indeed these patients can show various combinations of clinical, cognitive, behavioral, psychosocial, and environmental issues (2), which can interfere with the effectiveness of rehabilitation interventions. It has been proposed that the efficacy of rehabilitation should increase if programs would move from disease-centered to person-centered, tailored on the needs of every single individual (3, 4). In this way, it would be simpler to target routines, occupations, and relationships more effectively (5). The International Classification of Functioning, Disability and Health (ICF) suggests that goals, to be considered person-centered, must be set at the level of participation or contextualized in a life situation (1).

Generally, there is a lack of common measures to evaluate clinical outcome after rehabilitation treatments. Moreover, the variability between cognitive rehabilitation approaches involving multiple cognitive domains did not aid to understand whether and how this recovery can be generalized to everyday routine (home management, extra-domestic activities, social relationship, and productivity) (6).

Such improvements need to be defined in terms of autonomies, allowing healthcare professionals to tailor and apply specific rehabilitation plans (7). In the rehabilitation realm the concept of autonomy is built on the self-determination, the ability to make decision, while independence is considered the ability to act in accordance with one's wishes and to perform activities without help from others (i.e., self-reliance) (8, 9). Unfortunately, there are no objective outcome measures useful to disentangle these specific domains.

For this reason, in 2013 we created a new tool: the Progression of Autonomies Scale (PAS) (8), to define disability profiles in ABI patients submitted to rehabilitation. The PAS measures levels of autonomy in activities daily living (ADL), in domestic activities, and in the external environment supporting the design of individual rehabilitation plans targeting enhancement of autonomy. The PAS stems from the needs to define broad disability profiles in ABI patients submitted to rehabilitation treatment and to obtain a profile of an individual's autonomy over different domains, during the cognitive and behavioral rehabilitation process. The PAS is able to evaluate both the consequences of disability in terms of levels of autonomy in different domains and the progression of recovery over a wide range of activities, according to the biopsychosocial approach adopted by ICF (1). This scale is able to characterize the patient's autonomy and its progression over time through 3 different macrodomains: 1- Personal, 2- Domestic, and 3- Extra-domestic (8).

The aim of the present study was to evaluate the reliability of the PAS on a large cohort of ABI patients, providing additional information about the quality of measurements by means of the inter-rater agreement/reliability and intra-rater agreement/reliability, also referred to as test-retest (10).

## Materials and Methods

### Patients

From January 2017 to June 2018 we consecutively enrolled 51 ABI patients admitted in a dedicated rehabilitation unit of the S. Anna Institute of Crotone (Italy). Inclusion criteria were: (a) inpatient aged 16–65 years|; (b) severe ABI (Glasgow Coma Scale score ≤ 8; coma duration more than 3 days), (c) and informed consent by the proxy or surrogate to participate in this study. The exclusion criteria were: motor, cognitive or behavioral disabilities prior to ABI, and neurodegenerative brain diseases.

The study protocol was approved by the Ethical Committee of the University “Magna Graecia” of Catanzaro, according to the Helsinki Declaration. The patients' relatives and caregivers were informed about the experimental procedure and gave their written informed consent.

### The Progression of Autonomies Scale (PAS)

This scale is administered before the inpatient begins and at the end of a rehabilitation treatment, by a rehabilitation healthcare professional (occupational therapist, neuropsychologist, physical therapist, rehabilitation nurse). In both assessments, the professional observes (direct observation) the subject performing the activities required and assigns a score.

The same scale is also administered as a questionnaire at the admission to the patient -if health conditions allow him/her to answer the items-, and his/her caregiver, in order to verify their degree of awareness about his/her present disabilities, and again at the end of the rehabilitation program to define the improvements obtained and possible increases in the degree of patient and caregiver awareness. Comparing the 3 different scores assigned to each item (by the patient, the caregiver, and the healthcare professional), it is possible to determine how the single patient and caregiver perceive the disability. In particular, the target is to quantify how similar the patient and caregiver's evaluations are to the healthcare professional objective assessment, with regard to (i) the patient's basic functioning and autonomy before starting rehabilitation treatment, and (ii) the level of recovery at discharge.

The degree of mismatch detected can provide indications for planning the patient's rehabilitation treatment, as well as for optimizing the training and involvement of the caregiver, who is required to provide a certain degree of assistance in order to stimulate patient's autonomy recovery. Finally, one of the general goals of the rehabilitation team interventions should be to reduce, as far as possible, the mismatch between the evaluations among the caregiver, patient and healthcare professionals.

The healthcare professional assigns each item a score ranging from 0 to 3, where 3 indicates full autonomy and 0 indicates complete lack of autonomy. If the patient necessitates help from the caregiver, the scores 0 or 1 will be assigned; the scores 2 and 3 define the patients able to carry out the task autonomously (absence of a caregiver).

The final version of the PAS [38 items; (8)] was used to perform the evaluation of internal reliability.

#### Statistical Analysis

Two different blind expert health care professionals [an occupational therapist (A) and a neuropsychologist (B)] performed a clinical assessment with the PAS. Both raters have a wide background with brain-injured and disorders of consciousness patients, and were using the PAS for at least 4 years. The blind study was designed as follow: during the inter-rater agreement, both raters were present at the PAS administration. Only one administered the item, asking the patient to perform the required activity, but each one assigned independently the score, without any communication with the patient. Successively, each rater separately administered the PAS, in order to perform the intra-rater agreement. In addition to the usual PAS administrations (admission, monthly and discharge), an extra administration 10 days before the patient discharge was executed. This interval of time (10 days) was considered not significant for possible variation in terms of patients performance, but useful to evaluate the intra-rater agreement. The data resulting from the extra and final assessments were considered to be analyzed for the purpose of the intra-rater agreement. While raters assessments at the discharge were collected to be compared with the inter-rater agreement.

To measure the coefficient of agreement between and within raters the K Cohen test was applied (11). The level of agreement between raters and the level of consistency across the 2 times of assessment was estimated using the Kappa Cohen test. Kappa values were interpreted as: no agreement if *k* < 0; slight if 0 ≤ *k* ≤ 0.2; fair if 0.21 ≤ *k* ≤ 0.4; moderate if 0.41 ≤ *k* ≤ 0.6; substantial if 0.61 ≤ *k* ≤ 0.8; and almost perfect if 0.81 ≤ *k* ≤ 1 (12).

## Results

The study population included 51 ABI patients [36 male (mean age 46.1 ± 14.5) and 15 female patients (43.2 ± 11.3)], with different etiology (49% traumatic, 33.33% hemorrhagic, 17.64% other), at any time from the injury (mean time 456.6 days).

The data analysis of PAS scoring showed results ranging from substantial (0.61 ≤ *k* ≤ 0.8) to almost perfect (0.81 ≤ *k* ≤ 1) agreement for both inter-rater and intra-rater agreement (Table 1).

**Table 1:** K Cohen test results.

**Inter-rater agreement**				**Intra-rater agreement**	
**Extra assessment**	***k***	**Discharge assessment**	***k***		***k***
A_tot_ vs. B_tot_	0.66	A_tot_ vs. B_tot_	0.70	A_tot_-D vs. A_tot_-E	0.88
A_p_ vs. B_p_	0.69	A_p_ vs. B_p_	0.71	B_tot_-D vs. B_tot_-E	0.94
A_d_ vs. B_d_	0.92	A_d_ vs. B_d_	0.87	A_p_-D vs. A_p_-E	0.87
A_ed_ vs B_ed_	0.91	A_ed_ vs. B_ed_	0.85	B_p_-D vs. B_p_-E	0.94
				A_d_-D vs. A_d_-E	0.90
				B_d_-D vs. B_d_-E	0.98
				A_ed_-D vs. A_ed_-E	0.89
				B_ed_-D vs. B_ed_-E	0.99

*A, Rater A; B, Rater B; tot, total score; p, personal domain; d, domestic domain; ed, extra-domestic domain; D, discharge assessment; E, extra assessment*.

In the inter-rater agreement, the agreement degrees in the total scores found between raters A and B were *k* = 0.66 and *k* = 0.70 at the extra and discharge assessments, respectively. Increasing agreement degrees were detected in the subscores, both at the extra and discharge evaluations, referring to Personal (*k* = 0.69 and *k* = 0.71), Domestic (*k* = 0.92 and *k* = 0.87) and Extradomestic (*k* = 0.91 and *k* = 0.85) domains.

In the intra-rater agreement analysis, the very high results showed an almost perfect agreement. The extra vs. discharge assessments of each rater were compared. In the total score, the raters showed *k* = 0.88 (rater A) and *k* = 0.94 (rater B). In the subscores of each domain, the min and max agreement degree found for the rater A were *k* = 0.87 (Personal) and *k* = 0.90 (Domestic), while *k* = 0.94 (Personal) and *k* = 0.99 (Extradomestic) for the rater B, respectively.

## Discussion

The PAS is a well-structured tool assessing the autonomy levels of brain-injured patients. It provides a broad profile of the patient within multiple domains of functioning and meets the needs to monitor, quantify, and generalize the recovered abilities to the daily routine activities. Moreover, the assessment by caregiver and patient allows to stimulate the involvement of caregiver in the rehabilitation treatment and to increase the degree of awareness in the patient, respectively.

This study is aimed at testing the reliability of the PAS, by means of evaluation of the inter-rater and intra-rater agreement analysis performed in a different cohort of ABI patients with respect to our previous study (8). As above reported (see Table 1), high level of reliability of the PAS was revealed, with data ranging from substantial to almost perfect agreement for the inter-rater agreement and almost perfect agreement for the intra-rater agreement. This evidence confirms (8) that this tool is useful to assess the levels of autonomy in personal ADL, domestic activities and in the external environment. As the purpose is to measure the autonomy level in patients with residual disability (motor, cognitive), the PAS takes into account the contextual (environmental and personal) factors as defined by the ICF figuring out how to modulate the domestic environment in order to better regulate the activities and participation restrictions.

Despite the PAS is able to better characterize the autonomy levels in different kind of patients, to date its application was limited to ABI patients. Future directions should be the application to different healthcare settings and additional investigations about the relationship between score profiles, as defined by the questionnaire submitted to patients with respect to caregivers.

## Author Contributions

FA conceived and designed the study. FA, MC, and FR collected, analyzed, and interpreted the data. FA drafted the manuscript. AC and PT revised critically the manuscript. LFL, SS, and AM provided critical feedback. All authors approved the final version of the manuscript.

### Conflict of Interest Statement

The authors declare that the research was conducted in the absence of any commercial or financial relationships that could be construed as a potential conflict of interest.
